# Do Self-Objectified Women Believe Themselves to Be Free? Sexual Objectification and Belief in Personal Free Will

**DOI:** 10.3389/fpsyg.2019.01867

**Published:** 2019-08-08

**Authors:** Cristina Baldissarri, Luca Andrighetto, Alessandro Gabbiadini, Roberta Rosa Valtorta, Alessandra Sacino, Chiara Volpato

**Affiliations:** ^1^Department of Psychology, University of Milano-Bicocca, Milan, Italy; ^2^Department of Educational Sciences, University of Genoa, Genoa, Italy

**Keywords:** sexual domain, objectifying gaze, self-objectification, mental states, belief in free will

## Abstract

The present study aims to investigate the indirect link between sexual objectification and belief in personal free will. We hypothesized that being subjected to objectifying commentary would lead women to self-objectify and, in turn, to perceive themselves as having less personal free will. In this study, 105 women were asked to sign up a website created for this study by providing a personal description and a photo. After signing up, they received feedback from a fictitious male user. Depending on the condition, the comment was neutral (baseline), focused on their description (non-objectifying) or focused on their physical appearance (objectifying). The results showed that participants in the objectifying condition (vs. non-objectifying vs. baseline) self-objectified (i.e., perceived themselves as lacking human mental states and more as an instrument vs. a human) and, in turn, believed that they had less personal free will. The theoretical and practical implications of these findings for educators and therapists are discussed.

## Introduction

Valuing women on the basis of their sexual attractiveness rather than their skills is a pervasive tendency that still permeates most western societies. This focus on physical appearance affects women’s inner states, by leading them to self-objectify, i.e., to self-value and view as a mere body rather than a full human being. An emerging clear outcome of this state of self-objectification is that women are less prone to stand up against the unfair gender *status quo* and participate in collective actions aimed at challenging it (Calogero, 2017).

The main goal of the present work is to deepen the understanding of such pattern, by focusing on a specific human ability that may meaningfully contribute to explain the reasons for this undermined motivation. Thus, through an experimental study we analyzed whether sexual objectification – understood as the male focus on women’s physical appearance – and, in particular, women’s consequent self-objectification undermines the belief in personal free will, i.e., the perception of being able to make free and conscious decisions (Baumeister and Monroe, 2014).

### Sexual Objectification and Self-Objectification

Sexual objectification refers to the treatment and perception of women as mere objects (Nussbaum, 1995). In this process, the objectified person becomes, in the eyes of observers, an “*inhuman body*” (Vaes et al., 2014). This term aptly represents the two main dimensions defining objectification: *instrumentality* and *denial of humanness* (see also, Nussbaum, 1995). When women are objectified they are treated as mere bodies, instruments for the use and pleasure of others (*instrumentality*). Further, they are deprived of their personhood and considered as mindless entities, unable to experience human mental states (*denial of humanness*).

According to the Objectification Theory (Fredrickson and Roberts, 1997), the main means of objectification is the *objectifying gaze*, which refers to the more or less explicit male attitudes, sexual innuendos or comments focusing on women’s physical appearance. These types of interpersonal feedback can be internalized by women and trigger their self-objectification, that is their enhanced attention on their bodies and physical appearance rather than on their full person (Calogero et al., 2009).

Objectification and the related increased self-objectification lead to several negative psychological consequences (see Calogero et al., 2011). For example, Loughnan et al. (2017) found that recalling an objectifying situation leads women to see themselves as less human and less moral. Chen et al. (2013) found that objectification elicits sinful feelings in female victims. In their study, women believed that they were interacting with a male partner via an online chat and, depending on the condition, received comments focused on their physical appearance or on their general character. The results showed that comments about physical appearance led women to experience greater sinful feelings and a greater perception of dirtiness. In the same vein, Kahalon et al. (2018) found that receiving appearance-related compliments leads to lower cognitive performance.

Of particular interest for the present study, some recent research has also reported that self-objectification affects how women live and interact in social scenarios, by undermining their active social presence. For example, Saguy et al. (2010) showed that objectified women tend to limit their presence in dyadic interactions by speaking less when talking with a male partner. Calogero (2013) found that self-objectified women are more likely to endorse system justification beliefs (Jost et al., 2004). In turn, these beliefs predicted a reduction of their activism in supporting collective actions aimed at changing the sexist *status quo* (see also Calogero, 2017).

### Self-Objectification and Belief in Personal Free Will

The present study considers a further consequence of women’s self-objectification: the perception of having personal free will, that is the belief of being capable of making free and conscious choices (Baumeister and Monroe, 2014). This fundamental human dimension has different social functions as leads people to make decisions that improve oneself and one’s own situation (e.g., Baumeister, 2005; Vohs and Schooler, 2008; Baumeister et al., 2009; Feldman et al., 2014). However, belief in free will can be affected by different factors and its reduction is associated with, for example, greater mindless conformity to the opinions of others (Alquist et al., 2013) and a consequent possible acceptance of discriminating situations (Baumeister and Monroe, 2014). In light of this possibility, we argue that belief in free will could be a relevant variable when considering gender relationships in which women often occupy – and accept – disadvantaged positions (see also Calogero, 2017).

In particular, we hypothesized that women’s belief in personal free will could be affected by self-objectification. In doing so, we considered the dimensions of both instrumentality and lack of humanness. In fact, we argue that both of these dimensions are critically associated with a decreased belief in personal free will. First, the ability to exert free will is based on the fundamental human abilities of self-control and rational thought (Baumeister et al., 2011). Therefore, perceiving oneself as lacking humanness, and thus feeling unable to experience particular mental states (see Haslam et al., 2008) that form the basis for exerting free will, may lead women to believe that they have no free will. Second, perceiving oneself as an instrument or an objectified body – rather than as a human being – may affect one’s belief in personal free will, since objects are conceived as passive entities directed by external forces (Michotte, 1946; Dennett, 1987; Wegner, 2002). Initial support for these assumptions, albeit limited to the domain of work, has been provided by Baldissarri et al. (2017, 2019). The authors found that performing objectifying activities indirectly leads people to self-perceive as lacking personal free will through increased self-objectification, both in terms of self-perception as an instrument and as lacking humanness. In the present study, we aimed to verify this indirect link between objectification and reduced free will in the sexual domain.

To sum up our hypotheses, in accordance with previous research on sexual objectification (e.g., Chen et al., 2013; Kahalon et al., 2018), we first assume that being subjected to an objectifying gaze – i.e., male comments focused on physical appearance – may lead women to self-objectify, in terms of self-perception both as an instrument and as lacking human mental states. In turn, these self-perceptions may lead to a decrease in their belief of having personal free will.

## The Study

We tested our hypotheses in an experimental study in which sexual objectification was manipulated by giving female participants different types of fictitious feedback (similar to Chen et al., 2013). Specifically, participants were asked to sign up an ostensible undergraduates’ online community for sharing study notes. The website was built to create an ecological framework in which female participants, after signing up, received feedback from a senior male user. In the objectifying condition (vs. non-objectifying condition vs. baseline), the senior male user motivated the participant’ acceptance in the community by emphasizing her physical appearance (vs. skills vs. no motivation). Afterward, measures of self-objectification and belief in personal free will were assessed. Self-objectification was measured in terms of the two dimensions related to a self-view as *inhuman body:* self-perception as lacking human mental states and as increased perception of oneself as an instrument (vs. a human).

## Methods

### Participants and Experimental Design

Considering the smallest effect size of the manipulation of the objectification reported by Chen et al. (2013; Study 1, ${\text{η}}_{p}^{2}$ = 0.14), a power analysis was conducted with Gpower (ver. 3.1; Faul et al., 2009). The analysis suggested that a minimum sample size of 99 participants was needed for a large power (0.95). One hundred and five female undergraduate volunteers were recruited from a large university in northwester Italy. Participants ranged in age from 19 to 40 years (*M* = 23.81; *SD* = 3.09). All participants were Italian (except 1 Albanian). Participants were randomly allocated to one of three conditions (objectifying vs. non-objectifying vs. baseline condition).

### Procedure and Materials

Participants were individually examined. As a cover story, they were told that they would be asked to test the usability of a website for sharing study notes. Afterward, participants completed a questionnaire assessing the dependent variables of interest. Finally, participants were asked to provide demographic information and were thanked and fully debriefed.

#### The Website

**FIGURE 1:**
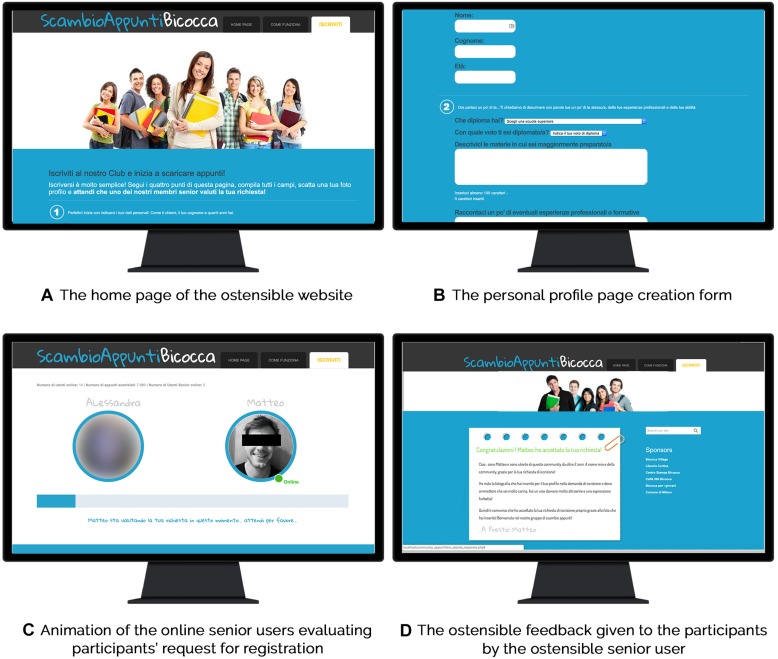
Screenshots of the ostensible website adopted for the manipulation. The fictitious male senior user was presented without covering black bars.

The website was built specifically for our experiment (Figure 1A). Participants were told that the website was a closed web community for sharing notes for university courses and therefore, after signing up, a senior user had to approve their application by evaluating their personal profile page. Similar instructions were also reported in the pages of the website. Participants were free to browse the website: they could open all available pages describing the website, the service, and the community. When ready, participants could sign up by creating their personal profile page (Figure 1B). In doing so, they were asked to introduce themselves by inserting a description of their studies history, their skills and training. To complete the subscription, participants were asked to snap and upload a picture with a webcam. After completing the registration, a screen in which they were asked to wait until one of the online senior users replied to their registration request appeared. After 45 s, a picture of a fictitious male senior user, named Matteo, appeared on the screen, communicating to the participants that he was evaluating their application to join the community (Figure 1C).

#### Feedback

After 2 min of waiting, feedback from the “senior user” appeared on the screen (Figure 1D). The feedback was fictitious, and its appearance on the screen was controlled by a computer script, and started with the following sentences: *“Congratulation [participant name]! Matteo has approved your request!*

Hi [participant name], I am Matteo, and I’ve been a user of this community for two years. On behalf of the community and me, thank you for your application.”

In the baseline condition, participants’ acceptance to the community ended in this way: “*I inform you that I have accepted your application for registration! Welcome to our study notes sharing community!”*

In the objectifying condition, the fictitious senior member motivated his choice by focusing on the uploaded photo with the following sentence: *“I have had a look at the picture you uploaded for your profile, and I have to say that you look very pretty, you have a very attractive face and a slyexpression! Therefore, I inform you that I have accepted your application for registration because of the picture you uploaded! Welcome to our study notes sharing community!”*

As for the non-objectifying condition, the user focused on the description provided by the participants*: “I have read the description you entered for your profile, and I have to say that you look very smart, the language you used isvery appropriate and you truly look qualified! Therefore, I inform you that I have accepted your application for registration because of the description you entered! Welcome to our study notes sharing community!”*

### Dependent Variables

#### Self-Objectification

The dimension of self-attribution of humanness was assessed by the *self-mental state attribution task* (SMSA; Haslam et al., 2008; Baldissarri et al., 2014). Participants were asked to rate (1 = not at all; 7 = very much) the extent to which they felt themselves able to experience 20 different mental states (α = 0.91) during the website experience. Mental states referred to perceptions (e.g., hearing), thoughts (e.g., reasoning), wishes (e.g., desiring), intentions (e.g., planning), and emotions (e.g., fear, pleasure). The *self-perception of being an instrument* was measured by adapting the measure of instrumentality used by Andrighetto et al. (2017) and asking participants to rate (1 = not at all; 7 = extremely) the extent to which they perceived themselves as similar to three instrument body-related items (α = 0.70; instrument, thing, body) and three human-related items (α = 0.86; human being, person, individual). The mean of the human-related scores was subtracted from the mean of instrument body-related scores to create a single index: higher scores indicate an increased self-perception of being an instrument (vs. a human).

#### Belief in Personal Free Will

We adopted the *personal will* subscale (8 items; α = 0.88) from the free will and determinism scale (Rakos et al., 2008) for assessing participants’ belief in personal free will. In particular, participants were required to state the degree to which they believed they had free will (1 = not at all; 7 = extremely) after the experience on the website. Sample items included “I am in charge of my actions even when my life’s circumstances are difficult,” “I actively choose what to do from among the options I have,” and “I have free will.”

#### Attentional Check

Participants were asked if the feedback received from the senior user was based on their physical appearance. Responses included *yes, no, do not remember*. Participants who replied incorrectly or did not remember (*N* = 6) were not considered in all the analyses. Thus, the considered final sample was composed of 99 participants.

## Data Analysis and Results

As shown in Table 1, participants’ ratings on the SMSA were positively correlated with belief in personal free will, whereas self-perception as an instrument (vs. a human) was negatively correlated with free will.

**TABLE 1:** Correlations between the measured variables.

**Variables**	**1**	**2**	**3**
1. SMSA	–		
2. Self-perceptions of being an instrument	−0.42^∗∗∗^	–	
3. Belief in personal free will	0.37^∗∗∗^	−0.35^∗∗∗^	–

**^∗∗∗^p* ≤ 0.001. SMSA, self-mental state attribution.*

A between-participants MANOVA was conducted to verify the extent to which the conditions affected the dependent variables (SMSA, self-perception as instrument-like, belief in personal free will). The multivariate test revealed a main effect of the condition, λ = 0.65*, F*(6,188) = 7.41, *p* < 0.001, ${\text{η}}_{p}^{2}$ = 0.19. As reported below, univariate tests showed a significant effect of condition on each dependent variable (see Table 2).

**TABLE 2:** Mean ratings of SMSA, self-perceptions of being an instrument (vs. a human) and belief in personal free will as a function of feedback manipulation.

	**Conditions**		
**Variables**	**Objectifying**	**Non-objectifying**	**Baseline**
SMSA	3.09_a_ (0.92)	4.37_b_ (0.94)	3.69_c_ (0.97)
Self-perceptions of being an instrument	−1.34_a_ (2.67)	−3.42_b_ (1.69)	−3.18_b_ (2.33)
Belief in personal free will	4.65_a_ (0.89)	5.49_b_ (1.17)	4.56_a_ (1.24)

*Means with different subscripts in the same row differ significantly, *p* < 0.05. Standard deviations are provided in parentheses.*

### Self-Objectification

For SMSA, the analysis showed a significant effect of the condition, *F*(2,96) = 15.18, *p* < 0.001, ${\text{η}}_{p}^{2}$ = 0.24. In particular, Bonferroni-adjusted comparisons indicated that the participants in the objectifying condition self-attributed fewer human mental states than participants in the non-objectifying (*p* < 0.001, *d* = −1.37) and baseline conditions (*p* = 0.033, *d* = −0.63). The mean scores in the non-objectifying condition differed from those of the baseline condition (*p* = 0.013, *d* = 0.71), indicating that participants in the non-objectifying condition self-attributed more human mental states than those assigned to the baseline condition. A similar pattern of results emerged for perceiving oneself as being an instrument, *F*(2,96) = 8.33, *p* < 0.001, ${\text{η}}_{p}^{2}$ = 0.15. Participants in the objectifying condition perceive themselves as more like an instrument (vs. a human) than participants in the non-objectifying (*p* = 0.001, *d* = 0.93) and baseline conditions (*p* = 0.004, *d* = 0.73), while the participants’ instrument mean scores in the baseline and non-objectifying conditions did not significantly differ (*p* = 1.00, *d* = −0.11).

### Belief in Personal Free Will

The univariate test for belief in personal free will showed a main effect of the condition, *F*(2,96) = 6.87, *p* = 0.002, ${\text{η}}_{p}^{2}$ = 0.12. In this case, participants in the objectifying condition believe that they have less personal free will than participants in the non-objectifying condition (*p* = 0.009, *d* = −0.79), whereas the difference with the baseline condition was not significant (*p* = 1.00, *d* = 0.08). Moreover, participants in the non-objectifying condition reported a significant increase in their belief in personal free will compared to those in the baseline condition (*p* = 0.003, *d* = 0.76).

**FIGURE 2:**
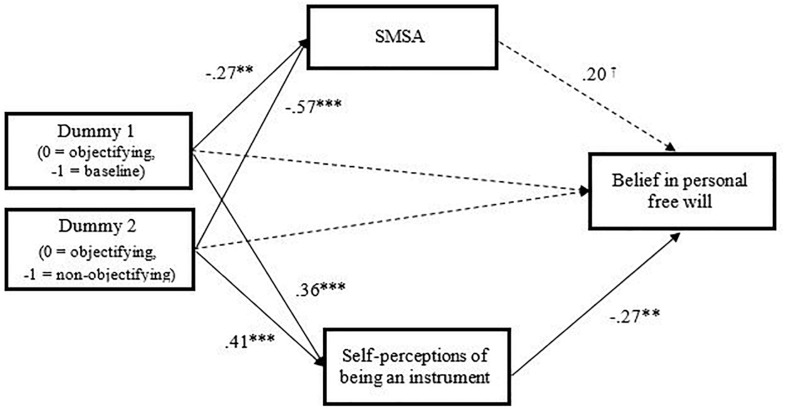
Conditional process model testing the indirect effect of the objectifying feedback (vs. non-objectifying feedback vs. baseline feedback) on the belief in personal free will via self-perceptions of being an instrument (vs. a human) and SMSA. The reported values are standardized beta coefficients. SMSA, self-mental state attribution. †*p* = 0.062, ^*^p ≤ 0.05, ^∗∗^*p* ≤ 0.01, ^∗∗∗^*p* ≤ 0.001.

To examine the prediction that receiving objectifying feedback (vs. non-objectifying feedback vs. baseline feedback) would indirectly decrease the belief in personal free will via SMSA and self-perceptions as an instrument (vs. a human), we conducted a conditional process model by considering the two self-objectification dimensions as mediators in parallel (Model 4 of the PROCESS macro for SPSS with 5,000 bootstrapping samples; Hayes, 2013; see Hayes, 2009). Furthermore, because the independent variable was categorical with three levels, we followed Hayes and Preacher’s (2014) recommendations by generating two dummy-coded variables with the objectifying condition as the reference group. As shown in Figure 2, the contrasts of the objectifying feedback vs. baseline feedback (D1) and of the objectifying feedback vs. non-objectifying feedback (D2) led to increased self-perceptions as instrument [D1: *b* = 1.84, *SE* = 0.56, *t*(2,96) = 3.30, *p* = 0.001; D2: *b* = 2.08, *SE* = 0.56, *t*(2,96) = 3.73*, p* < 0.001] and to a decrease in SMSA [D1: *b* = −0.60, *SE* = 0.23, *t*(2,96) = −2.59, *p* = 0.011; D2: *b* = −1.28, *SE* = 0.23, *t*(2,96) = −5.51*, p* < 0.001]. In turn, the increase in self-perceptions as instrument (vs. human) led to participants’ decreased belief in personal free will [*b* = −0.13, *SE* = 0.05, *t*(4,94) = −2.66, *p* = 0.009], while the decrease in SMSA was not significantly related to this belief [*b* = 0.22, *SE* = 0.12, *t*(4,94) = 1.89, *p* = 0.062]. Furthermore, when considered together with the dimensions of self-objectification, the direct effect of D1 and D2 on belief in personal free will was non-significant (respectively, *p* = 0.092 and 0.369). Finally, the indirect effects of the conditions on the decreased belief in personal free will via SMSA were not significant (*a* × *b* = −0.13, 95%CI [–0.38, 0.002] for D1, *a* × *b* = –0.29, 95%CI [–0.75, 0.05] for D2). However, confirming our hypothesis, the indirect effects of the experimental condition on the decreased belief in personal free will via self-perceptions as instrument (*a* × *b* = −0.24, 95%CI [−0.66, −0.03] for D1 and *a* × *b* = −0.27, 95%CI [−0.65, −0.07] for D2) were significant (for alternative conditional process models see the Supplementary Material).

## General Discussion

By employing an ecological website paradigm, our findings show that experiencing objectifying situations (i.e., receiving comments focusing on the physical appearance) leads women to self-objectify, both in terms of decreased self-attribution of human mental states and increased self-perception of being an instrument rather than a human. These self-objectifying perceptions, in turn, lead to a decrease of their belief in personal free will.

Such a belief has been an unexplored outcome of self-objectification in the sexual domain so far. However, we believe that belief in personal free will is a relevant dimension to better comprehend many gender interactions. These relationships are often characterized by asymmetrical power, in which women are subordinate and not prone in engaging activities to change the *status quo* (Calogero, 2017). We believe that our study represents an attempt to elucidate a possible mechanism by which sexual (self-) objectification might contribute to undermine women’s intention to engage in active actions. Indeed, it could be that women, when experiencing self-objectification, are less inclined to rebel against dangerous relationships because of a decreased belief in personal free will. Accordingly, such experience could affect women’s tendency to “say no” in particularly ambiguous situations and to engage in active actions of rebellion. From this perspective, our finding also supports the idea that sexual objectification consists of a transformation of women in entities lacking “the human rights of wellbeing and freedom” (Le Moncheck, 1985. p. 2, as cited in Zurbriggen, 2013). These reflections are in line with recent studies that explored the impact of appearance compliments on people’s performance (Kahalon et al., 2018). As Kahalon and colleagues argued, such compliments, as they are commonly and specifically addressed to women, represent a subtle mechanism that perpetuates gender inequality, by undermining not only women’s performance, but also their beliefs to have the ability to make free choices.

About the emerged pattern of findings it is noteworthy that, although our main hypothesis was confirmed, our results show that the objectifying male gaze does not directly affect participants’ belief in personal free will. On one hand, women treated in an objectifying way reported similar levels of belief in personal free will to those who received neutral feedback. This lack of difference may suggest that some forms of objectification, in this case a comment on women’s physical appearance, are so common and widespread that they do not directly affect their beliefs regarding free will. On the other hand, our findings revealed that when female participants received a comment positively emphasizing their personal competence (i.e., non-objectifying condition), it increased their belief in personal free will, at least if compared to neutral feedback. This unexpected finding may suggest interesting new paths. Indeed, it is plausible to hypothesize that, for women, the attribution of personal competence can have an opposite effect to that of objectifying comments, by increasing their self-perception of being powerful agents feelings and their empowerment.

Regarding the causal path between self-objectification and belief in personal free will, the conditional process model confirmed the hypothesis that self-objectification, conveyed through objectifying feedback, affects this peculiar belief. However, it is noteworthy that when considering the objectifying vs. non-objectifying feedback (D2) in the alternative models (see the Supplementary Material), belief in personal free will mediated the relationship between receiving objectifying feedback and increased self-objectification. Therefore, it is plausible to hypothesize a bidirectional relationship between self-objectification and belief in personal free will, in which the perception of having less free will could, in turn, strengthen the women’s self-perception of being similar to an object and thus contribute to create a vicious detrimental circle.

Furthermore, the multiple measures that we used to assess self-objectification can expand the methodological knowledge for research on sexual objectification. In fact, the measures usually employed in the sexual objectification domain focus mainly on body surveillance or on the perceived importance of the body (e.g., objectified body consciousness scale, McKinley and Hyde, 1996; self-objectification questionnaire, Noll and Fredrickson, 1998). In the present study, we focused on the specific dimensions related to self-perception as an *inhuman body*: self-perception as a mere instrument body and self-perception as an inhuman entity. With regard to the importance of these two dimensions, our results suggest that self-perception of being an instrument has a stronger impact on women’s belief in personal free will than decreased self-attribution of human mental states. In fact, the conditional process model revealed that the indirect effect of objectifying condition on belief in personal free will via SMSA was not significant, at least when it was considered together with self-perceptions of being an instrument. It is noteworthy that when we tested a conditional process model considering only SMSA,^1^ the effect of SMSA on belief in personal free will was found to be significant as the indirect effects from the independent variable through the SMSA. Therefore, it may be that the presumably high portion of variance shared with self-perception of being an instrument and its predominant role could explain the null effect of SMSA in the model presented here.

### Limitations and Future Directions

Despite the novelty of our findings, it is important to acknowledge some limitations that may restrict their generalizability and interpretation.

First, the experimental paradigm we have developed for manipulating objectification was focused on a specific experience of sexual objectification (i.e., receiving positive feedback about physical appearance) in a specific context (an online community). Future research should replicate our findings by considering different experiences of sexual objectification in different contexts.

Second, future studies should consider the effects of feedback provided by senior female users. Given that objectifying comments make salient the focus on physical appearance regardless of the gender of the commenter, we would expect similar negative effects. However, some evidence shows, for example, that women exposed to female gaze report fewer negative consequences than those exposed to male gaze, suggesting that comments by other women might be perceived more positively, as providing a sort of social support (see Calogero, 2004). Future research should disentangle this interesting issue.

Third, future research should individuate possible moderators intervening in the proposed pattern. For example, it could be interesting to verify whether women’s initial levels of self-sexualization would shape the impact of appearance comments on their belief in free will. We would expect that high (vs. low) self-sexualized women, at least at an explicit level, would perceive these comments as empowering rather than objectifying and thus benefit from these comments, also in terms of increased belief in free will. Furthermore, it would be important to verify whether our pattern of findings would also emerge among different samples. In particular, women in more disadvantaged positions (e.g., unemployed women) may report a different pattern due to the limited possibilities of choices that characterize their lives, which may in turn negatively affect their tendency to make active choices (Stephens et al., 2011).

Finally, our study did not consider behavioral outcomes possibly predicted by decreased belief in personal free will. That is, a more exhaustive picture of the entire psychological process could be obtained by verifying whether indeed this decreased belief leads self-objectified women to be less willing to stand up against discriminatory and aggressive male behaviors, such as sexual harassment.

### Practical Implications

We believe that our findings have practice implications that can be of interest for multiple professionals, as they provide further evidence supporting the idea that specific conditions can inhibit women from “saying no.” Indeed, we showed that when women self-objectify when subjected to an exterior objectifying gaze, they had a reduced belief in their ability to make autonomous and free choices. In countries like Italy, where media are hyper-sexualized (e.g., Zanardo, 2010; Valtorta et al., 2016), the daily objectifying gaze that women face can negatively affect the choices and options that they perceive they have. Therefore, our findings can be useful for activists and promoters of movements aimed at sensitizing women and men about the possibilities to make free choices under critical conditions. Furthermore, therapists who support harassed women should focus on strengthening their belief in personal free will, which may have been undermined by processes of objectification and self-objectification.

## Conclusion

Findings of our work suggest that women, who are objectified daily and consequently are pushed to objectify themselves, could feel incapable of making conscious choices, and thus be less able to rebel when placed in these situations.

Given the importance and the sensitivity of these issues for today’s societies, we hope that our study can encourage future research to more deeply investigate the critical relationship between sexual self-objectification and women’s belief in personal free will.

## Data Availability

The dataset for this study is available through the Open Science Framework (https://osf.io/u5y3b/?view_only=75c553ca82124b92bfeeb47d3184bb02). The design and analysis plans were not preregistered.

## Ethics Statement

This study was carried out in accordance with the APA ethical guidelines with written informed consent from all participants. All participants gave written informed consent in accordance with the Declaration of Helsinki. Ethical approval was at the time of data collection not required by the Institution’s guidelines and national regulations, as the research was not of a medical nature and there were no potential risks to the participants.

## Author Contributions

CB, LA, RV, AS, and CV contributed to the conception and the design of the study. AG was responsible for the development of the paradigm and website. CB, LA, RV, and CV were responsible for the data collection, analysis, and interpretation of data. CB wrote the manuscript with valuable inputs from the remaining authors. All authors agreed for all aspects of the work and approved the version to be published.

## Conflict of Interest Statement

The authors declare that the research was conducted in the absence of any commercial or financial relationships that could be construed as a potential conflict of interest.

## Footnotes

1 In the conditional process model considering only SMSA, the effect of SMSA on belief in personal free will was found to be significant [*b* = 0.32, *SE* = 0.12, *t*(3,95) = 2.76, *p* = 0.007] as the indirect effects from the independent variable through the SMSA (D1: *a* × *b* = −0.19, 95%CI [–0.49, −0.04]; D2: *a* × *b* = –0.41, 95%CI [–0.87, −0.05]).
