# Supplementary material for: Do Self-Objectified Women Believe Themselves to Be Free? Sexual Objectification and Belief in Personal Free Will
Source: Front Psychol. 2019 Aug 8;10:1867. doi: 10.3389/fpsyg.2019.01867 (PMC6694764; doi:10.3389/fpsyg.2019.01867)
Supplement: Supplementary file 1 [file Presentation_1.pdf]

SUPPLEMENTARY MATERIAL

Original Italian feedback

Baseline condition: *"Ti comunico che ho accettato la tua richiesta di iscrizione! Benvenuta nel nostro gruppo di scambio appunti!"*

Objectifying condition: *"Ho visto la fotografia che hai inserito per il tuo profilo nella domanda di iscrizione e devo ammettere che sei molto carina, hai un viso davvero molto attraente e una espressione furbetta! Quindi ti comunico che ho accettato la tua richiesta di iscrizione proprio grazie alla foto che hai inserito! Benvenuta nel nostro gruppo di scambio appunti!"*

Non-objectifying condition: "*Ho letto la descrizione che hai inserito per il tuo profilo nella domanda di iscrizione e devo ammettere che sei molto intelligente, il linguaggio che hai utilizzato è molto appropriato e sembri veramente una persona preparata! Quindi ti comunico che ho accettato la tua richiesta di iscrizione proprio grazie alla descrizione che hai inserito! Benvenuta nel nostro gruppo di scambio appunti!"*

Alternative Conditional Process Models

A series of alternative models considering belief in personal free will as the mediating variable and both self-perceptions as an instrument (vs. a human) and SMSA as the dependent variables were also tested. For SMSA, the indirect effect of D2 was significant ($a*b$ = -.19, 95% CI [–.46, -.03]), but the indirect effect of D1 was not ($a*b$ = -.02, 95% CI [–.09, .20]). Furthermore, in this model, D1 and D2 still maintained their direct effect on belief in personal free will when entered together with the mediator (b = -0.63, SE = 0.23, $t(3, 95)$ = -2.77, p = .007 for D1 and b = -1.09, SE = 0.24, $t(3, 95)$ = -4.63, p < .001 for D2). A similar pattern resulted for self-perceptions as instrument. Therefore, competitive models suggested a mediation of belief in personal free will only for one of the dummies that, however, maintained its effect on the dependent variables. Thus, results

supported our proposed model in which the indirect effect of both the dummies on belief in personal free will via SMSA and self-perceptions as instrument resulted significant.
